# Broad Support Among Stakeholders for Collaboration Between Traditional Bonesetters and Formal Healthcare: A Qualitative Study in a Resource-Limited Setting

**DOI:** 10.1177/00469580251325031

**Published:** 2025-03-27

**Authors:** Joost Binnerts, Thom C.C. Hendriks, Nneka Buzugbe, Jovine Okoth, Carolina Torres Perez-Iglesias, Nefti Bempong-Ahun, Geoffrey Ibbotson, William J. Harrison, Claude Martin, Michael Edwards, Erik Hermans, Bwire Chirangi

**Affiliations:** 1 Radboud University Medical Centre, Nijmegen, The Netherlands; 2 Stichting Shirati, Amsterdam, The Netherlands; 3 Vrije Universiteit Medical Centre, Amsterdam, The Netherlands; 4 Shirati KMT Hospital, Shirati, Mara Region, Tanzania; 5 Harvard Medical School, Boston, MA, USA; 6 Global Surgery Foundation, Geneva, Switzerland; 7 AO Alliance, Davos, Switzerland

**Keywords:** intersectoral, collaboration, orthopedics, traditional, bonesetter, medicine, fracture, injury, trauma, health system, qualitative research, focus group discussion, interviews, resource-limited settings, Tanzania

## Abstract

Extremity fractures are increasingly common in Sub-Saharan Africa. In many resource-limited settings, patients with fractures have historically sought out traditional bonesetters (TBSs) and continue to do so, in part due to the undercapacity of existing orthopedic facilities. This qualitative study investigates key stakeholder perspectives on intersectoral collaboration between the formal healthcare system and TBSs in treating extremity fractures in the Rorya district, Tanzania. We combined focus group discussions and semi-structured interviews with four key stakeholder groups: patients with previous fractures, TBSs, hospital staff, and local government representatives. Questions concerned stakeholder experience, advantages of TBS and hospital care, perspectives on collaboration, and potential facilitators and/or barriers. Transcripts were analyzed using thematic analysis and inductive coding. Between June 2022 and August 2023, 35 TBSs, 9 patients with previous fractures, 5 hospital staff members, and 2 government representatives were interviewed. Participants unanimously recognized the need for collaboration between TBSs and hospitals. Identified barriers included TBSs’ motivation for hospital referral, poor customer care at hospitals, and limited understanding of fracture management in hospitals by TBSs and patients. Implementation of a collaborative triage and referral system was most commonly suggested. This study summarized all relevant perspectives on intersectoral collaboration. A combined approach of a joint triage and referral system, augmented by community education and TBS training, may enhance the quality and accessibility of fracture care and potentially serve as a model for regions facing similar challenges. Further research is needed to evaluate the feasibility and effectiveness of such initiatives in practice.

**Highlights**

● Hospital staff, fracture patients, government workers and traditional bonesetters unanimously agree on intersectoral collaboration

● Collaborative triage and referral system is the most frequently suggested model

● Potential facilitators to collaboration include incentives, respect for trade secrets and viewing each other as equal parties

## Introduction

Between 1990 and 2015, the rate of road traffic injuries more than doubled in 15 Sub-Saharan African countries, including Tanzania.^1^ Orthopedic injuries, particularly fractures, constitute a major part of the consequences of these injuries. However, there is limited availability of orthopedic clinical providers to address the growing burden, especially in rural areas. A study by Premkumar et al estimated that 90% of the population in Northern Tanzania does not have access to orthopedic surgical care.^2^

In Tanzania, 60% to 70% of the population meets their healthcare needs through traditional medicine,^3,4^ which the World Health Organization defines as “*the sum of the knowledge, skills, and practices based on the theories, beliefs, and experiences indigenous to different cultures, whether explicable or not, used in the maintenance of health and the prevention, diagnosis, improvement or treatment of physical and mental illness*.”^5^ Within this sector, traditional bonesetters (TBSs) commonly provide care for patients with extremity fractures in rural areas(Agarwal & Agarwal, 2010). In the study by Sudi and Muthanje, traditional bonesetting (TBS) is defined as “*health practices, approaches, knowledge and beliefs incorporating plant, animal and mineral-based medicines, spiritual therapies, manual techniques, and exercises, applied singularly or in combination to diagnose and treat fractures in the human bod*y.”^6^ Studies from Nigeria and India showed the preference for TBSs is driven by cultural acceptability, increased affordability, and geographical convenience.^7-9^

While TBSs may relieve the disease burden on the formal healthcare sector, concerns exist regarding the complications associated with TBS treatment.^10-12^ In 2010, Agarwal and Agarwal suggested that adequate training in orthopedic essential care provision may reduce these complications, allowing TBSs to play an important role within the formal healthcare sector.^13^ A more recent study by Konadu-Yeboah et al in Ghana showed that formal TBS training effectively improved TBS knowledge on fracture management and suggested that a network linking TBSs to the formal care system may increase potential patient referral from TBSs to the hospital.^14^

To establish such a collaboration, key stakeholders’ perspectives on efficient collaboration between the formal and informal healthcare sector are essential. Previous research shows TBSs’ willingness to collaborate through receiving training and access to allopathic techniques, including anesthesia, fixation materials, and X-ray imaging.^15-17^ However, these studies often focus on a single stakeholder group with small sample sizes, while governmental representation is lacking. Therefore, this study aims to provide insight into the perspectives of diverse stakeholders on the hospital care-based and TBS models, and establish the level of support for collaboration between these two models. Additionally, it explores stakeholder views on potential challenges and barriers toward such a collaboration. This could help guide future policy and ensure its acceptability among targeted populations.

## Methods

### Local Context

**Figure 1.**
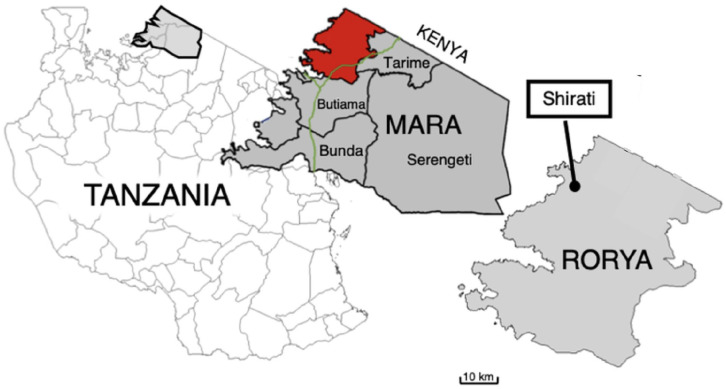
Rorya district map, Tanzania. Adapted from: Geisen, W. R., Bartone, C., Gerdes, D., & Lewis, C. (2021). Figure adapted with permission of the authors.

This qualitative study was conducted in the Rorya district, Northern Tanzania, as this area is renowned for its high patronage of traditional bonesetters due to its rurality (Figure 1). In 2022, the Rorya district had a population of 354 490 inhabitants,^18^ which is served by a single district hospital in Shirati, two health centers and dispensaries in most villages. The district hospital is a 5-h journey by car to the nearest referral hospital and employs 7 general doctors, receiving an orthopedic specialist for 1 week twice yearly. Despite governmental efforts, no exact data are available on the number of traditional bonesetters in Rorya.

### Study Design

This conventional qualitative study conformed to the Standards for Reporting Qualitative Research (SRHR) guidelines, to obtain an overview of the perspectives from the local formal healthcare system and the informal TBS system and the way they view local fracture care. We define the formal healthcare sector as a structured system that involves licensed professionals and institutions, which follow standardized practices and regulations. We employed a combination of focus group discussions and semi-structured interviews. These individual interviews were added to create a safer environment for participants to admit to treatment complications, as potential power dynamics between the TBS participants may occur during the focus groups, where they might feel uncomfortable sharing this with more senior peers out of shame. The field team consisted of JO, a male research assistant, NB, a female medical student and JB, a male medical doctor.

### Study Participants

To achieve triangulation, four key stakeholder groups were identified. In this study, we involved:

-Patients with previous extremity fractures, who had completed fracture treatment at a hospital, a TBS, or at both settings
-District hospital staff with experience in fracture care (e.g. doctors, nurses)
-Traditional bonesetters
-Government employees responsible for health policy in the Rorya district

Exclusion criteria for participants were:

-Patients aged <18 years, as the type of healthcare sought is generally decided by caretakers
-Patients with previous fractures of the chest, spine, neck, or skull
-Patients with fractures treated through any other method than hospital care or traditional bonesetting
-Government employees, hospital staff and TBSs with <1 year work experience

Potential participants were approached face-to-face to participate through a combination of convenience and snowball sampling. Patients and TBSs living in the same village were approached for a focus group discussion, to understand the local informal health system best. Individual bonesetters in scattered villages across the Rorya district were approached for semi-structured interviews, to get an overview of TBS perspectives in the entire district. A government community health worker assisted in contacting the TBSs. Informed consent for participation in the study was obtained from all participants through written consent before the interviews and focus groups. All questions regarding the study were answered prior to consenting.

### Data Collection

In June and October 2022, two focus groups were conducted for patients with previous fractures and TBSs. For the locations of both focus groups, a neutral setting for both interviewers and focus group participants was provided. The TBS focus group comprised 15 participants and was conducted in a primary school classroom. The patient focus group, involving 9 participants, was conducted in a hotel conference room in Shirati. Both focus groups lasted approximately 2.5 hours and were conducted in Kiswahili by JO, a Tanzanian investigator. A second foreign investigator (JB), proficient in Kiswahili, was present as an observer.

Between November 2022 and August 2023, individual key informant interviews with TBSs, hospital staff, and local government representatives were performed. Face-to-face semi-structured interviews with TBSs were conducted at their respective homesteads. The duration of the interviews was between 25 and 60 minutes. Interviews were conducted in Kiswahili or Kijaluo by JO.

Interviews with hospital staff and government employees lasted between 20 and 40 minutes and were conducted in English by NB, with a second Tanzanian investigator, JO, present. Hospital staff members were interviewed at the hospital in a private office, while the interviews with government staff members were conducted at private local government offices. Inclusion took place until data saturation was reached.

### Data Collection Tools

Specific interview and focus group guides were developed for each stakeholder (see Supplemental Appendix A). These were then checked and modified with the help of local colleagues. No formal pilot with participants was conducted to avoid priming the study’s included participants, particularly the TBSs. General themes were stakeholder experience with fracture care, perceived advantages of TBS and hospital care, support for intersectoral collaboration, and potential facilitators/barriers to success.

Audio during the interviews and focus groups was recorded using the native voice-recording application of a Tecno Spark 20 mobile phone and a Samsung S8 mobile phone. In addition, audiovisual footage of the focus groups was recorded using a Canon EOS 1000d camera. Field notes were also written down during data collection. Subsequently, two researchers (NB and JB) separately worked on verbatim transcriptions of all audio records using Microsoft Word, finding consensus on a final version afterward. In case of persisting disagreement, a third researcher (TH) made the final decision. If the audio record was in Kiswahili or Kijaluo, translation into English was done at the same time as transcription. The transcripts of the focus groups and the key informant interviews were de-identified and stored digitally in MAXQDA Pro 2022. Participants were offered access to the audio recording and transcript. Active returning of transcripts was not done. After data analysis, findings were discussed with participants during local results dissemination. This did not influence the study’s conclusions.

### Data Analysis

The transcripts were analyzed through inductive coding,^19^ using the framework method for thematic analysis of the retrieved data.^20^ All transcripts were reviewed by NB and JB. Two separate codebooks were then created through open coding using the data analysis software of MAXQDA. Consensus was then reached on a final codebook. A third analyst (TH) made the decision if no consensus was reached. Subsequently, indexing was performed on the generated codes, creating clusters of codes that shared commonalities or consistencies, representing the different stakeholders’ perspectives. A code system was thus generated, in which 4 main themes were identified:

1. Patient and Community Perceptions of Healthcare Providers
2. Access to Healthcare Services
3. Need for Collaboration
4. Forms of Collaboration

These themes and their specific subthemes are described in the Results section. A full overview of the code hierarchy and frequencies can be found in Supplemental Appendix B.

### Ethics Statement

Ethical approval of the study was obtained from the National Institute for Medical Research Tanzania.

## Results

During the study period focus groups were conducted with 15 TBSs and 9 patients. Interviews were conducted with 20 TBSs, 5 hospital staffers and 2 government workers. There was no previous relationship between the research team and the participants. One TBS participant was excluded from the study because they declined to be audio-recorded during the interview. One repeat interview with a TBS was carried out, due to loss of the original audio recording.

Demographic data on the study participants is shown in Table 1. Among TBSs, men and women were similarly represented. Most considered themselves Christian, which reflects the general population in Rorya district. The median age was 57 years, and the median work experience 12 years. Monthly income earned as TBS was a median 75.000 TSH, or $28. Patients with previous fractures were generally younger at a median of 30 years. Nearly all patients were male and Christian. Patient level of education ranged from primary school to college. Most had been treated at the hospital at some point, though half of that group had also been treated by a TBS. The hospital staff members all worked in fracture care, with a median 18 years of work experience.

**Table 1.** Overview of participant characteristics.

Traditional bonesetters	35 (100%)
*Median age (N = 27)*	57 years
*Sex (N = 35)*	
Male	19 (54.3%)
Female	16 (45.7%)
*Level of completed education (N = 27)*	
None	5 (18.5%)
Primary school	22 (81.5%)
Secondary school	0 (0%)
*Religion (N = 28)*	
Christian	23 (82.1%)
Muslim	5 (17.9%)
*Median work experience (N = 30)*	12 years
*Median monthly income as TBS (N = 24)*	75.000 TSH
Past fracture patients	9 (100%)
*Median age (N = 9)*	30 years
*Sex (N = 9)*	
Male	8 (88.9%)
Female	1 (11.1%)
*Religion (N = 9)*	
Christian	8 (88.9%)
Muslim	1 (11.1%)
*Level of completed education (N = 9)*	
Primary school	1 (11.1%)
Secondary school	6 (66.7%)
College	2 (22.2%)
*Mode of treatment (N = 9)*	
Hospital care	4 (44.4%)
Traditional bonesetting	1 (11.1%)
Both	4 (44.4%)
Hospital staff	5 (100%)
*Median age (N = 5)*	40 years
*Sex (N = 5)*	
Male	3 (60%)
Female	2 (40%)
*Religion (N = 5)*	
Christian Muslim	4 (80%) 1 (20%)
*Occupation (N = 5)*	
Doctor	1 (20%)
Nurse	3 (60%)
Physiotherapist	1 (20%)
*Median work experience*	18 years

## Patient and Community Perceptions of Healthcare Providers

### Traditional Beliefs on Bonesetting

Two main pathways were identified for becoming a TBS: either through an apprenticeship with more experienced TBSs, or through inheritance of skills of elders. For the latter group, ancestral spirits were reported to guide TBSs in executing their skills through dreams. Some bonesetters reported that a combination of apprenticeship and inheritance is also possible. One hospital staff member added that after receiving treatment for a prolonged period at the TBS, some patients become TBSs themselves without additional training. After becoming a bonesetter, recognition within communities was often gained through word of mouth.

“. . . it’s like giving the heritage, and then after that it takes a few days until he (the old bonesetter) dies. And after that, the spirits come to the chosen person to instruct him through dreams.” (Bonesetter 5, interview)

### Perceptions of TBSs

When discussing the advantage of TBS, the frequent follow-up of fracture patients was an aspect strongly valued. Patients also often considered fracture healing at the TBS to be quicker than at the hospital. In additional, multiple TBSs and one hospital staff member cited the significant influence of relatives on healthcare decisions, who often prefer TBSs due to positive previous experiences and the TBS’ respected position in the community.

A limitation of TBS treatment often described by hospital staff members and patients was the limited prevention against infections, resulting in impaired wound healing or disability. Given examples concerned multiple aspects of wound care, including not administering tetanus prophylaxis when indicated, not performing surgical debridement and unsterile treatment of the fracture site by using bare hands instead of gloves.

Several TBSs acknowledged that a lack of access to gloves was a challenge often faced when treating patients. One TBS reported that, prior to treatment, he sent each patient to undergo hospital screening for tuberculosis and HIV to prevent potential spread of disease.

“The first challenge we are facing is lack of equipment like gloves. We are doing this job in a risky environment.” (Bonesetter 8, interview)

Several patients reported that pain management during fracture reduction or massage and physiotherapeutic exercise opportunities were often insufficient when treated by a TBS due to the lack of equipment, materials and medications. Many TBSs agreed pain management was a challenge during treatment.

“But our main challenge is when you’re massaging the client, they feel such great pain. . . We only use herbs for massaging. We don’t have pills to reduce pain. We need help with that.” (Bonesetter 9, focus group)

Hospital staff agreed unanimously on the perceived lack of familiarity with patient triage procedures among TBSs. TBSs were reported to have limited access to essential diagnostic tools, including X-rays and laboratory tests, that are crucial for effective triage. Several staff members linked these limitations to both acute complications, such as hypovolemia, and long-term complications, including non-union and deformities.

### Perceptions of Hospital-Based Care

The possibility of surgical intervention was identified as an advantage of hospital-based treatment, as acknowledged by the majority of hospital staff members and six TBSs. Bonesetters acknowledged their deficiency in crucial surgical skills and the absence of requisite surgical equipment, which occasionally led them to advise patients to continue fracture treatment at a hospital.

“. . . when the patient came, I advised him to go to the hospital first for check-up, because sometimes you may find the surgery is needed and I don’t have skills and tools to do that.” (Bonesetter 4)

Regarding conservative management, one hospital staff member explained that patients often do not understand the effectiveness of the use of plaster casting and think that the frequent treatment interval of TBSs contributes more to fracture healing. Many TBSs agreed that the unpopular use of Plaster of Paris at the hospital contributes to patients’ preference for treatment by a TBS.

“Patients think that if you just put the Plaster of Paris, nothing is happening. Which means the people, they don’t know that the healing of the bone is taking place by itself. . . So, whenever they’re being touched (by a bonesetter), maybe palpating, that’s where they know that that man is doing what is needed in the bone, which is not true.” (Hospital staff member 1, interview)

“The patients would complain, since at times the bandages and plaster would have lice in them. That was uncomfortable and would only add to their pain. The traditional way, once you apply the herbs, there is no way for the lice to get to the patients.” (Bonesetter 16)

Another frequently mentioned limitation of hospital care was the perceived poor quality of customer service in comparison to TBSs. Many patients explained that waiting times at the hospital can be extensive. One staff member added that these waiting times frequently lead to patients leaving the hospital early in favor of seeking treatment from a TBS. Furthermore, patients reported to have experienced unprofessional language and communication during their hospital interactions. Poor customer care was also mentioned by TBSs to be a limitation of hospital treatment. Finally, participants from each stakeholder group concurred that fear of amputation was a significant factor, deterring people from seeking hospital care.

## Access to Healthcare Services

### Financial Barriers

Patients, TBSs, government officials, and most hospital staff members agreed that treatment at the TBS was more affordable than hospital care. However, one hospital staff member added that this does not apply for fracture patients who develop complications during treatment at the TBS, who consequently require expensive surgical management. According to nearly all TBSs, high hospital bills often form a challenge for patients, leading patients to discontinue hospital treatment and opting for TBS treatment instead.

“. . . This (high hospital bills) is why they are running to us. Here we do not do it as a business. We are concerned with the wellbeing of a person first, rather than making a profit.” (Bonesetter 2, interview)

Bonesetter treatment costs ranged from 30 000 to 200 000 Tanzanian Shilling (Tsh, or 11-75 dollar), depending on given treatment and treating bonesetter. Starting fees were reported to be between 10 000 and 50 000 Tsh. After treatment, patients were generally asked to make a final payment, mostly depending on the duration of stay. Some TBSs claimed they did not take any treatment costs into account. Two TBSs either accepted whatever patients offered or adjusted the treatment costs to the patient’s economic status.

“It (the treatment cost) depends on a person’s condition. We differ economically, so I just receive according to the patient’s capacity.” (Bonesetter 4, interview)

Many bonesetters providing care without charging, or at low costs, did so since they perceived their skills to be a gift of God, aiming to serve the community.

“We receive anything as a symbol of gratitude from patients. (. . .) We don’t take this job as a business. Rather, it’s for helping people because we are given the skills by God freely.” (Bonesetter 2, interview)

Non-monetary payments, including food and cattle, were also accepted by several TBSs. According to some hospital staff members, patients often preferred this over the monetary payments required at the hospital. The flexibility of paying in installments during and even after TBS treatment was also considered an advantage.

### Geographical Barriers

A majority of hospital staff members viewed the geographic distance between patients and the hospital as the primary challenge for hospital care. However, 1 staff member contended that distance was not the sole determinant, as some fracture patients residing in close proximity to the hospital still opted for traditional bonesetting. None of the participants from other stakeholder groups actively noted distance as a factor.

“The distance is not so much a problem, because even those (patients) who are near to the hospital, they are also leaving (to the bonesetter). . .” (Hospital staff member 5, interview)

### Healthcare Infrastructure

The majority of bonesetters acknowledged the presence of specific criteria requiring hospital referral and treatment. These criteria included cases demanding tetanus prophylaxis (6%), open fractures (26%), femoral fractures (12%), fractures of the hand (3%), multifragmentary fractures (9%), fractures with severe malalignment (6%), polytrauma (3%), older fractures with mal- or non-union(3%), and non-traumatic fractures (3%). All hospital staff members reported that initial treatment should start at the hospital when a fracture is classified as open, fragmented or poorly aligned. Most staff members (80%) agreed that TBS treatment can be sufficient for the treatment of closed extremity fractures, providing correct alignment.

Multiple staff members reported a difference in expertise between bonesetters. TBSs’ opinions were partially in line with this perspective, since they reported having an informal referral system within their network of bonesetters. When cases exceeded the scope of their treating abilities or the progress was not as expected, some TBSs opted to either refer to or consult a more experienced bonesetter within their network, who was considered to be of a higher level. Two TBSs acknowledged the existence of “scamming” bonesetters, who might continue treating complicated patients for profit.

“Of course, even us we have shortcomings like we have false colleagues, scammers, that are stealing from patients. . .” (Bonesetter 2, interview)

Referrals from bonesetter to hospital occurred less frequently than referrals to fellow bonesetters. TBSs tended to refer patients to the hospital primarily for the diagnosis of a potential fracture and for follow-up using X-ray examination. Only three bonesetters reported to never have referred a patient to the hospital, explaining that their treatment had never failed.

“You must know when to stop, you can’t pretend that you can treat everything.” (Bonesetter 11, focus group)

## Need for Collaboration Between Hospitals and TBSs and Potential Challenges

### Need for Collaboration

When queried on the need for collaboration between hospitals and TBSs, unanimous consensus emerged among hospital staff members, TBSs, government representatives, and patients.

“Yes, there is a need, because the main objective for both of us is to help the patient to recover. So, by cooperating, it will be a great thing and useful for patient wellbeing.” (Bonesetter 5, interview)

“It’s a big need. People are having troubles out there and some, some even having deformities without reason. . .” (Hospital staff member 4, interview)

### Facilitators and Barriers to Collaboration

In previous collaborations in maternal care, traditional birth attendants were reportedly offered incentives for referring patients to the hospital. Where government officials were in favor of providing similar incentives to referring TBSs, hospital staff members held varying opinions regarding this matter. While a portion of the staff advocated for offering financial incentives to TBSs, others posited that incentives should be extended only in the form of education, provision of food, or covering transportation expenses incurred by TBSs. TBSs themselves indicated that incentives in the form of monetary compensation or educational opportunities could serve as appropriate motivations.

Lack of respect for trade secrets, such as herbal mixture ingredients, was also mentioned to be a factor attributing to previous collaborations not having succeeded. One TBS suggested that respecting these trade secrets would facilitate future collaboration.

“So many people have used this as the opportunity to steal from us and even there were times that the foreigners came for the purpose of knowing the medicines and what disease they treat, only to take our skills. How can we cooperate with such people?” (Bonesetter 8, interview)

Some TBSs and a hospital staff member emphasized that respect and acknowledgment for traditional bonesetting in general would facilitate a future collaboration.

“We are not acknowledged, so that makes us not want to refer clients to the hospital immediately.” (Bonesetter 11, focus group)

## Forms of Collaboration

### Collaboration Pathways

All hospital staff members advocated for the implementation of a triage system, to differentiate between those fit for treatment by TBSs and those needing hospital care. The proposed triage system would primarily rely on X-ray imaging for diagnosis. Several patients were also in favor of undergoing X-ray investigations before any form of treatment. Most staff members and government representatives were convinced that uncomplicated fractures with well-aligned fracture parts could, following X-ray evaluation, be suitable for treatment by TBSs.

“I think it will be good if we cooperate, and from that we can identify types of patients that we can manage and for the hospitals to manage also.” (Bonesetter 1, interview)

Lastly, proponents among both TBSs and patients proposed a collaborative model in which TBSs and medical staff would work in tandem at the hospital when managing fracture patients. To achieve this, it was suggested that a shared workspace within the hospital be created.

“I think the hospital should make a collaboration with these people (traditional bonesetters), because their aim is that the patient recovers. So maybe we can simplify the procedure for patients by researching if they truly help patients recover and adopt them to be part of the hospital. . .” (Patient 1, focus group)

### Community Education

The majority of staff members believed that community health workers and village leaders, due to their significant local influence, played a crucial role in patient education concerning fracture treatment and should be engaged in future collaboration.

Among hospital staff members, a health educational campaign for patients and TBSs was frequently proposed, educating them on hospital referral criteria. The favored method for achieving this educational outreach predominantly involved conducting village-based outreach programs. TBSs themselves exhibited a receptive attitude toward trainings, aimed at facilitating knowledge and skill sharing.

### Regional and Governmental Collaboration

The local government employees interviewed appeared open to the idea of a collaborative system, based on equal terms and standardized guidelines.

“They may be thinking that in the partnership, the hospital would take advantage and take away their clients and patients. So, we. . . will guarantee them that they will continue with their work as usual, their income will come in as usual and that the hospital will also have their own.” (Government representative 2, interview)

## Discussion

The burden of road traffic accidents and associated extremity fractures in resource-limited settings remains high and is expected to increase. It has been estimated that 10% to 40% of fractures are treated by traditional bonesetters.^21^ To bridge this gap, several studies have proposed intersectoral collaboration, linking informal care with formal healthcare.^15,22^ This study is among the first to establish a fundamental understanding of the perspectives of all key stakeholders on sustainable collaboration between formal and informal healthcare in treating extremity fractures in a resource-limited setting. It expands on the body of evidence showing support for intersectoral collaboration and offers a pathway through which this may be achieved. Many of the perspectives highlighted in this study, such as the importance of financial incentives, respect, and equality as facilitators for collaboration, reflect universal truths, suggesting our findings may be helpful to clinicians in similar rural and resource-limited settings worldwide.

Stakeholders unanimously recognized the need for collaboration between TBSs and hospitals for the treatment of extremity fractures, though the suggested forms varied. A commonly suggested approach was a collaborative triage and referral system, utilizing hospital diagnostic options to establish which patients are suitable for traditional bonesetting and which patients require hospital treatment.

To this cause, key barriers identified include TBSs’ reluctance to refer patients to hospitals and the limited understanding of hospital fracture care among patients and TBSs. Proposed facilitators included offering a compensation for referring bonesetters, educating both community members and TBSs, reducing waiting time at the hospital and creating a shared working environment at the hospital.

While our study found strong support for collaboration among all included stakeholders, existing literature presents mixed views from TBSs. Studies from Nigeria suggested resistance due to spiritual beliefs and the perception that allopathic doctors encroach on their business.^23-25^ Conversely, studies done by Yempabe et al (Ghana) and Hancock et al (Chad) showed nearly unanimous support for collaboration (96.4 and 100%, respectively).^16,17^

A Nigerian survey by Asa et al suggested that a 63% majority of TBSs supports integration of informal care into the national health system, as well as 86.7% of orthopedic surgeons and 82.7% of patients.^26^ More recent studies reported that 75% to 100% of hospital staff favored collaboration with TBSs.^16,27^ These high levels of support for collaboration among patients and hospital staff are in line with this study’s findings. Since no previous studies have explored government perspectives on linkage with informal fracture care, the positive stance of the representatives in this study may not be generalizable.

In our study, several stakeholders agreed that patients preferred TBS treatment over hospital care for fear of amputation. This perception, reported in previous literature,^28-31^ may be mitigated through health education on fracture treatment. Unlike the suggestion made by Aderibigbe et al to solely highlight the dangers of TBS treatment,^28^ a balanced and respectful educational approach that objectively discusses both traditional and formal healthcare may be more successful.

But while TBS training may mitigate misconceptions and TBS-associated complications, a more structural approach may be necessary to establish a sustainable collaboration between TBSs and the formal healthcare sector. Previous successes in integrating traditional birth attendants into formal obstetric care in several African countries suggest that such collaborations are possible and beneficial.^32,33^

Similar to the results in our study, Kwame in Ghana supported establishing a shared unit for hospital staff and TBSs at the hospital, to facilitate information sharing and integration of medical innovations, such as X-ray imaging.^34^ Our study adds to this by highlighting the need for financial and non-financial incentives to motivate TBS referrals, drawing parallels with the positive impact of monetary incentives observed during the integration of traditional birth attendants.^35-37^ Additionally, similar to van der Watt et al, participants in our study emphasized that the key to successful collaboration is an egalitarian and respectful working relationship.^38^

### Recommendations

We strongly recommend establishing a collaboration between TBSs and the formal healthcare system for treating extremity fractures in rural and resource-limited settings with high TBS patronage. Geographical mapping of TBSs in the region should be conducted, combined with interviews to assess willingness and suitability among TBSs to join the collaboration program. This program should include a triage and referral system based on patient and fracture characteristics, with clear guidelines on which cases require hospital treatment and which can be managed by TBSs. To promote sustainability, a respectful professional relationship between medical staff and linked bonesetters should be established. A pilot collaboration should be conducted to identify and address practical barriers toward feasibility.

### Limitations and Strengths

A limitation of this study was the presence of a foreign researcher during the focus groups and individual interviews, which may have led to social desirability bias. To mitigate peer influence on responses, individual interviews were conducted in addition to focus groups. Although interviews were conducted at homesteads of TBS to improve safety, the vicinity of household members could have influenced their answers. Additionally, translation from Kiswahili into English could have reduced the reflection of participants’ views, despite requiring agreement between 2 translators. Finally, the interview guides were internally developed and thus not validated previously. A pilot was also not done to avoid priming of traditional bonesetters. However, this may have influenced validity of the guides.

## Conclusion

This study highlights the critical need for intersectoral collaboration between traditional TBSs and hospitals for treating extremity fractures in rural Tanzania. Identified key barriers and facilitators for effective collaboration include TBSs’ motivation for referral, understanding of hospital treatment among patients and TBS, and customer care and financial accessibility of hospital care.

We propose implementing a collaborative triage and referral system, based on patient and fracture characteristics, offering incentives for TBS referrals and promoting community and TBS education on fracture management. This combination of interventions has the potential to significantly improve healthcare outcomes in areas with high bonesetter patronage, by harnessing the strengths of both traditional and formal healthcare sectors.

## Supplemental Material

sj-docx-1-inq-10.1177_00469580251325031 – Supplemental material for Broad Support Among Stakeholders for Collaboration Between Traditional Bonesetters and Formal Healthcare: A Qualitative Study in a Resource-Limited Setting Supplemental material, sj-docx-1-inq-10.1177_00469580251325031 for Broad Support Among Stakeholders for Collaboration Between Traditional Bonesetters and Formal Healthcare: A Qualitative Study in a Resource-Limited Setting by Joost Binnerts, Thom C.C. Hendriks, Nneka Buzugbe, Jovine Okoth, Carolina Torres Perez-Iglesias, Nefti Bempong-Ahun, Geoffrey Ibbotson, William J. Harrison, Claude Martin, Michael Edwards, Erik Hermans and Bwire Chirangi in INQUIRY: The Journal of Health Care Organization, Provision, and Financing

sj-docx-2-inq-10.1177_00469580251325031 – Supplemental material for Broad Support Among Stakeholders for Collaboration Between Traditional Bonesetters and Formal Healthcare: A Qualitative Study in a Resource-Limited Setting Supplemental material, sj-docx-2-inq-10.1177_00469580251325031 for Broad Support Among Stakeholders for Collaboration Between Traditional Bonesetters and Formal Healthcare: A Qualitative Study in a Resource-Limited Setting by Joost Binnerts, Thom C.C. Hendriks, Nneka Buzugbe, Jovine Okoth, Carolina Torres Perez-Iglesias, Nefti Bempong-Ahun, Geoffrey Ibbotson, William J. Harrison, Claude Martin, Michael Edwards, Erik Hermans and Bwire Chirangi in INQUIRY: The Journal of Health Care Organization, Provision, and Financing

sj-pdf-2-inq-10.1177_00469580251325031 – Supplemental material for Broad Support Among Stakeholders for Collaboration Between Traditional Bonesetters and Formal Healthcare: A Qualitative Study in a Resource-Limited Setting Supplemental material, sj-pdf-2-inq-10.1177_00469580251325031 for Broad Support Among Stakeholders for Collaboration Between Traditional Bonesetters and Formal Healthcare: A Qualitative Study in a Resource-Limited Setting by Joost Binnerts, Thom C.C. Hendriks, Nneka Buzugbe, Jovine Okoth, Carolina Torres Perez-Iglesias, Nefti Bempong-Ahun, Geoffrey Ibbotson, William J. Harrison, Claude Martin, Michael Edwards, Erik Hermans and Bwire Chirangi in INQUIRY: The Journal of Health Care Organization, Provision, and Financing
