# Supplementary material for: Broad Support Among Stakeholders for Collaboration Between Traditional Bonesetters and Formal Healthcare: A Qualitative Study in a Resource-Limited Setting
Source: Inquiry. 2025 Mar 27;62:00469580251325031. doi: 10.1177/00469580251325031 (PMC11948548; doi:10.1177/00469580251325031)
Supplement: sj-docx-1-inq-10.1177_00469580251325031 – Supplemental material for Broad Support Among Stakeholders for Collaboration Between Traditional Bonesetters and Formal Healthcare: A Qualitative Study in a Resource-Limited Setting [file sj-docx-1-inq-10.1177_00469580251325031.docx]

## Appendix A – Focus group & interview guides

**Focus group questions: fracture patients**

1. How long after your fracture or you started experiencing pain did you seek fracture treatment?
2. Where did you go for fracture treatment?
3. Why did you choose this type of fracture treatment?
   - If non-response, suggest: expense, transport, recommendation from friend / family / relative
4. Were you happy with the fracture treatment you received?
5. If you could change anything about the treatment you received, what would it be? Why?
6. What do you think are the advantages of fracture management at the hospital? Why?
7. What do you think are the challenges of fracture management at the hospital? Why?
8. What do you think are the advantages of fracture management at the TBS? Why?
9. What do you think are the challenges of fracture management at the TBS? Why?
10. Do you think that the hospital and TBSs should start collaborating?
    - To those answering ‘yes’: why and in what way should the hospital and TBSs collaborate? For example: seek out each other’s advice only, work together for every fracture patient, practice in the same clinic
    - To those answering ‘no’: why do you feel the hospital and the TBSs should not collaborate?
11. Whether or not you think the hospital and the TBS should collaborate, what barriers do you see for efficient collaboration?
12. If you were to set up a collaboration between the hospital and TBSs, what aspects would you be sure to include in that collaboration?
13. Is there anything else you’d like us to specifically know about your fracture experience?

**Key informant interview questions: traditional bonesetters**

1. Could you briefly tell me about your work as a TBS?
2. Do you have any experience working together with healthcare workers?
3. What do you think about perspectives of healthcare providers towards TBSs?
4. Do you refer patients to the healthcare facilities? Why/why not? If so, which type of patients?
5. What fracture types would you consider necessitating ‘surgical treatment’?
6. If you have a patient with a fracture that you feel necessitates ‘surgical treatment,’ what would be your plan of action (or next steps)? Why? Have you ever had to do this?
7. What do you think are the advantages of fracture management at the hospital? Why?
8. What do you think are the challenges of fracture management at the hospital? Why?
9. What do you think are the advantages of fracture management at the TBS? Why?
10. What are the challenges you experience treating patients with a fracture? Can you tell me more about that?
11. What are things you would like to improve about the fracture treatment you give?
12. Do you think the hospital and TBSs should start collaborating on fracture treatment?
    - To those answering ‘yes’: why and in what way should the hospital and TBSs collaborate?
      If non-response beyond simply yes, prod with discussion of different collaboration options and get specific feedback on those collaboration pathways
    - To those answering ‘no’: why do you feel the hospital and the TBSs should not collaborate? Do you have any alternative ideas instead of collaboration between Hospitals and TBSs that could improve patient outcomes?
13. Regardless of if you think they should collaborate, what potential barriers do you see for efficient trauma care?
14. If you were to set up a collaboration between the hospital and TBSs, what aspects would you be sure to include in that collaboration?
15. Is there anything else you’d like us to specifically know about your experience as a TBS?

**Key informant interview questions: local government officials and hospital staff**

1. Do you know of any collaboration between health facilities and TBSs?
2. What’s your perspective towards TBSs and the work they do?
3. What do you think are the advantages of going to the hospital with a fracture? Why?
4. What do you think are the challenges of going to the hospital with a fracture? Why?
5. What do you think are the advantages of going to the TBS with a fracture? Why?
6. What do you think are the challenges of going to the TBS with a fracture? Why?
7. Do you think the hospital and TBSs should start collaborating on fracture treatment?
   - To those answering ‘yes’: why and then in what way should the hospital and TBSs collaborate?
     If non-response beyond simply yes, prod with discussion of different collaboration options (perhaps from the literature) and get specific feedback on those collaboration pathways
   - To those answering ‘no’: why do you feel the hospital and the TBSs should not collaborate?
   - Do you have any alternative options instead of collaboration between Hospitals and TBSs that could improve patient outcomes?
8. Regardless of if you think they should collaborate, what barriers do you see for efficient collaboration?
9. If you were to set up a collaboration between the hospital and TBSs what aspects would you be sure to include in that collaboration?
10. Is there anything else you’d like us to specifically know about your experience with fracture patients?
