# Supplementary material for: Broad Support Among Stakeholders for Collaboration Between Traditional Bonesetters and Formal Healthcare: A Qualitative Study in a Resource-Limited Setting
Source: Inquiry. 2025 Mar 27;62:00469580251325031. doi: 10.1177/00469580251325031 (PMC11948548; doi:10.1177/00469580251325031)
Supplement: sj-pdf-2-inq-10.1177_00469580251325031 – Supplemental material for Broad Support Among Stakeholders for Collaboration Between Traditional Bonesetters and Formal Healthcare: A Qualitative Study in a Resource-Limited Setting [file sj-pdf-2-inq-10.1177_00469580251325031.pdf]

Code System	Frequency
Code System	1094
Particulars of TBS care	0
Becoming a bonesetter	34
Cost of TBS treatment	23
Fracture diagnosis	4
Feeling	10
Non-touching	0
Spirits	5
X-ray	20
Interpretation	8
General treatment	4
Antibiotics	1
Fixation	14
Herbal treatment	22
Massage	16
Non-touching treatment	2
Pain treatment	13
exercise	3
Healing time	2
Monthly number of patients	25
Open fracture treatment	13
Other practiced types of traditional medicine	9
Perspectives on TBS treatment (+)	26
Religion	0
Animism	23
Christianity	13
Spiritual treatment	6
TBS registration	21
TBS treatment location	24
Time before fracture treatment	9
Years of treatment experience	17
provided care: non-medical interventions	8
Referral system	11
Bonesetter - bonesetter	14
Consulting another TBS	0
Bonesetter - hospital	28
Motives	12
Hospital - bonesetter	8
Hospital versus TBS	0
Bonesetter limitations	10
Client not willing to pay	19
Continuity of service	4
Lack of diagnostics	12
Lack of surgical equipment	10
Limited facilities at TBS	3

Longer healing time	1
Mode of communication	1
Painful	13
Permanent disability (+) (+)	24
Protection against disease	14
Quality of bonesetting	9
Scammers	2
Stigma on traditional healers	4
lack of infrastructure	1
Distance	0
Distance to nearest health facility	3
Patients distance to treating TBS	10
Hospital limitations	8
Amputation	8
Distance	9
Expensive	32
Monetary payment	4
Pain management	3
Patient understanding of conservative treatment	1
Poor communication	1
Poor customer care	16
Waiting time before care	7
Patient motives for TBS treatment	4
Customer care	2
Good previous experience at TBS	1
Influence of relatives/friends	10
Longer healing time hospital	11
Respected in the community	6
Use of POP	10
Patient motives for hospital treatment	33
Treatment indication	5
Hospital indication	43
TBS indication	19
Collaboration	4
Benefits	3
Challenges	11
Facilitators/barriers	3
Clear diagnostic protocols	1
Community involvement	6
Frequent communication	4
Funding of patient transportation	2
Incentives (financial/training)	12
Involving CHW	6
Respect/friendliness	3
Timely care	2
Need	48

Previous experience	58
Communication	3
Meetings	6
Traditional birth attendents	6
Suggested collaboration	58
Integration into hospital care	2
Referral system	2
Training and outreach (+)	11
Anatomy/physiology	0
Community	5
First aid	1
TBS	9
Triage of fractures/patient selection	5
X-ray interpretation	2
Training and outreach	0
X-ray interpretation	0
Triage of fractures/patient selection	0
Anatomy/physiology	0
First aid	0
